# Detection of dangerous events on social media: a critical review

**DOI:** 10.1007/s13278-022-00980-y

**Published:** 2022-10-22

**Authors:** M. Luqman Jamil, Sebastião Pais, João Cordeiro

**Affiliations:** 1 Department of Computer Science, University of Beira Interior, Covilhã, Portugal; 2 NOVA LINCS, New University of Lisboa, Lisboa, Portugal; 3 Groupe de Recherche en Informatique, GREYC, University of Caen Normandie, Caen, France

**Keywords:** Dangerous events, Social media, Event detection, Sentiment analysis, Extremism, Social network services, Social computing, Terrorism

## Abstract

The usability of the events information on social media has been widely studied recently. Several surveys have reviewed the specific type of events on social media using various techniques. Most of the existing methods for event detection are segregated as they approach certain situations that limit the overall details of events happening consecutively on social media while ignoring the crucial relationship between the evolution of these events. Numerous events that materialize on the social media sphere every day before our eyes jeopardize people’s safety and are referred to by using a high-level concept of dangerous events. The front of dangerous events is broad, yet no known work exists that fully addresses and approaches this issue. This work introduces the term dangerous events and defines its scope in terms of practicality to establish the origins of the events caused by the previous events and their respective relationship. Furthermore, it divides dangerous events into sentiment, scenario, and action-based dangerous events grouped on their similarities. The existing research and methods related to event detection are surveyed, including some available events datasets and knowledge-base to address the problem. Finally, the survey is concluded with suggestions for future work and possible related challenges.

## Introduction

The influence of social media on people’s lives and attitudes has been widely studied and established in many different perspectives (Messaoudi et al. 2022; Fu et al. 2020). Although social media is a broad term, it mainly refers to Facebook, Twitter, Reddit, Instagram, and YouTube. There are 4.66 billion active internet users worldwide, and 4.2 billion users are active on social media. As of the first quarter of 2020, Facebook has 2.6 billion monthly active users globally, making it the most extensive social media network globally. Twitter is one of the leading social media with 397 million users worldwide, becoming increasingly prominent during events and an essential tool in politics (Department 2021). Another study (Kwak et al. 2010) shows that Twitter is an effective and fast way of sharing news and developing stories. This trend has continued to grow over the last decade as the internet has become widespread. However, the use of social media has become more complex in the last decade. It became a broader phenomenon because of the involvement of multiple stakeholders such as companies, groups, and other organizations. It played a significant role in different outcomes, affecting countries, people, and the world. One such example is “Arab Spring” (Khondker 2011), an event that started in Tunisia and spread among other regional countries. Another example of good and bad events in the UK and US political spheres is given in the study that uses Twitter to evaluate the perceived impact on users (Moutidis and Williams 2020).

The recent example of violence in Bangladesh can explain the link between social media with real life. On Wednesday, 15 October 2021, clashes were sparked by videos and allegations that spread across social media that a Qur’an, the Muslim holy book, had been placed on the knee of a statue of the Hindu god Hanuman. The violence continued in the following days, which resulted in the deaths of 7 people, with about 150 people injured; more than 80 special shrines set up for the Hindu festival were attacked. This case shows social media’s severe and robust effect on our daily lives and ground situation (Wikipedia 2021). This violence was termed as “worst communal violence in years” by New York Times. Similar episodes of violence are becoming a norm in India since the rise of right-wing politics. If there is a prediction of such events or detection at an initial stage, it can alert the possible coming hazards to authorities. Such dangerous events can be countered in anticipation while significantly reducing the response time of authorities while maximizing the protection of people at risk.

The term “event” typically implies a change, an occurrence bounded by time and space. In the context of social media, an event can be happening on the ground/online or in a specific term. Different mediums can broadcast events happenings on the ground while people participate in the event through social media discussion. These kinds of events can be referred to as hybrid events (Bailo and Vromen 2017). While some events solely happen online, such as gaming, marketing, and learning events. The events-related discussion on social media reveals sentiments and opinions of the general public and the direction where the events are evolving. This quick interaction of users and transmission of information makes it a dynamic process that sometimes proves hard to follow the latest development, making it a challenging task. Event detection is a vast research field, and various requirements and challenges exist for each task. Various terms have been used to address different events, making it complex to navigate the literature. Most of the research in this field is segregated as different terminologies referring to the same thing, sometimes adding great misunderstandings.

This work introduces the term “Dangerous Events” that have a common root for various events and can be explained as a hyponym for dangerous. The dangerous events are divided into three main categories based on specific similarities and characteristics: sentiment-based, scenario-based, and action-based dangerous events. This division also helps us establish certain features necessary to link with other events and their evolution with time. As such, an event from a sentiment-based dangerous event can lead to a scenario-based and then to an action-based dangerous event and vice versa. Approaching the problem broader can help us formalize the technique to detect all the relevant dangerous events. Detecting all dangerous events and ranking them in the order of seriousness can help us save extra time and effort by detecting these events separately. This approach can help authorities detect and intercept such events while ensuring public safety and order.

The organization of this paper is as follows: Sect. 2 presents the definition related to dangerous events in social media. Section 3 reviews event detection methods and techniques; Sect. 4 discusses the dangerous event detection and event prediction with possible challenges. Section 5 presents the conclusion with some possible future research directions.

## Dangerous events

According to Merriam-Webster (2021), the word “dangerous” means involving possible injury, pain, harm, or loss characterized by danger. In that context, we define a dangerous event as the event that poses any danger to an individual, group, or society. This danger can come in many shapes and intensities. The objective is to draw a fine line between normal, harmless, unpleasant, extreme, abnormal, and harmful events. Less sensitive, unpleasant, and disliked events do not compel the person to feel threatened. While, in the case of dangerous events, the person will feel fearful, unsafe, and threatened. This provides the objective to approach the term “event” in a broader sense to address the common element of all such events. The details of dangers can always be discussed in detail, providing the necessity of the situation; for example, a natural disaster proceeds urgent hate speech. In other words, the first requires an immediate response with no time to lose, while the latter can allow some time to take action.

Dangerous events can be anomalies, novelty, outliers, and extreme. These terms can be used to refer to positive or negative meanings. However, not all anomalies, novelties, and extremes are dangerous, but all dangerous events fulfil one or all of those conditions (extreme, anomaly, novelty). Authors in Pais et al. (2020) proposed an unsupervised approach to detect extreme sentiments on social media. Positive Extreme sentiments can be detected and differentiated from everyday positive sentiments. Therefore, it may be concluded that extreme negative sentiments will likely turn into dangerous events.

Grouping and defining dangerous events based on their characteristics is another challenging task, and it can help address the issue of approaching different types of dangerous events by narrowing it down to specific details. We will define three broad categories of dangerous events with commonality among them.

1. Scenario-based Dangerous Events
2. Sentiment-based Dangerous Events
3. Action-based Dangerous Events

**Fig. 1:**
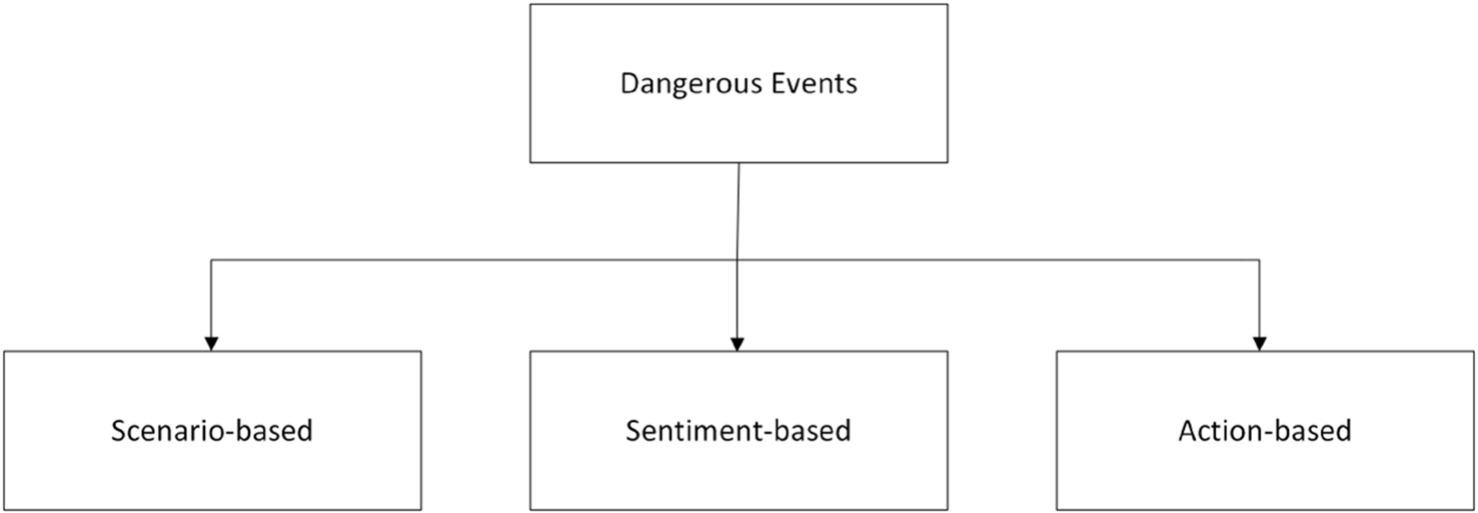
Dangerous events and their categories

Figure 1 depicts dangerous events and their categories. In the following subsections, we will outline the definition for each type of dangerous event.

### Sentiment-based dangerous events

Emotion is a complex psychological state such as fear, anger, or happiness while sentiment is a mental attitude produced by feelings. Sentiment and emotion are often used interchangeably. However, emotions are raw, while sentiments are organized. The sentiment can be thought, opinion, judgment, feeling, or emotion held or expressed towards a person, group, or entity. Regarding sentiment-based dangerous events, it can be any sentiment that poses a danger or can lead to a dangerous scenario or action. Distinct approaches have been proposed for text sentiment detection by researchers. Some of the commonly based methods include “Keyword-based”, “Lexicon-based”, “Machine-learning”, and “Hybrid” methods. Transformer-based models such as BERT are widely used for the detection of sentiments from different perspectives.

Sentiment Analysis (SA), also known as Opinion Mining (OM), is the process of extracting people’s opinions, feelings, attitudes, and perceptions on different topics, products, and services. The sentiment analysis task can be viewed as a text classification problem as the process involves several operations that ultimately classify whether a particular text expresses positive or negative sentiment (Geddes et al. 2015). For example, a micro-blogging website like Twitter is beneficial for predicting the index of emerging epidemics. These are platforms where users can share their feelings which can be processed to generate vital information related to many areas such as healthcare, elections, reviews, and illnesses. Previous research suggests that understanding user behaviour, especially regarding the feelings expressed during elections, can indicate the outcome of elections (Chandra and Saini 2021).

Sentiments can be positive and negative, but for defining sentiment-based dangerous events, the applicable sentiments are negatives and, in some instances, negative extremes such as hate speech, cyber-bullying, threats, anger, protest, antisemitism, islamophobia, xenophobia and extremism. Online radicalization can be attributed to this threat related to extreme negative sentiments towards certain people, countries, and governments. Such extreme negative sentiments can result in protests, online abuse, and social unrest. A prime example of dangerous sentiment can be the hate projected toward someone to inflict harm on someone. The critical factor behind dangerous scenarios and actions are mainly extreme negative sentiments that develop and manifests in the physical realm. Detecting these events can help reduce their impact by allowing the concerned parties to counter beforehand. A hypothetical example of a sentiment-based dangerous example of a tweet obtained using SocialNetCrawler is given below:

RT @Lrihendry: When Trump is elected in 2020, I’m outta here. It’s a hate-filled sewer. It is nearly impossible to watch the hateful at...

### Scenario-based dangerous events

We refer to the word “scenario” as the development of events. These events are unplanned and unscripted, and most of the time, they occur naturally. Some planned events can also turn into surprising scenarios. For example, a peaceful protest can turn into a riot, like in 2020 when a peaceful protest against corona restrictions in Germany turned into an ugly situation when the rally was hijacked by right-wing extremists, which ended up storming Parliament building and exhibiting right-wing symbols and slogans (Euronews 2020).

Detecting and tracking natural disasters on social media have been investigated intensively, and studies (Dwarakanath et al. 2021) have proposed different methods to identify those disasters by various means. The aim of these studies has been mainly to tap into the potential of social media to get the latest updated information provided by social media users in real time and identify the areas where assistance is required. This paper considers scenario-based dangerous events, including earthquakes, force majeure, hurricanes, floods, tornadoes, volcano eruptions, and tsunamis. Although each calamity’s nature is different, the role of social media in such events provides a joint base to approach them as scenario-based dangerous events. A supposed example of scenario-based danger is obtained using the crawler tool SocialNetCrawler, which can be accessed using the link^1^:

@politicususa BREAKING: Scientists predict a tsunami will hit Washington, DC on 1/18/2020 We Are Marching in DC... https://t.co/3af4ZhyV3J

### Action-based dangerous events

The action involves human indulgence in an event. Various actions happen on the ground that can be detected using social media. Actions can be of many types, but we point out actions that are causing harm, loss, or threat to any entity, which again shares the common attribute of negativity and is highly similar to previously defined types of dangerous events. Some action-based dangerous events include prison breaks, terrorist attacks, military conflicts, and shootings. Several studies have been published focusing on one or more types of such action-based events. The study (Lenihan 2022) focuses on anti-fascist accounts on Twitter to detect acts of violence, vandalism, de-platforming, and harassment of political speakers by Antifa. An assumed example of an action-based example is given below:

RT @KaitMarieox: This deranged leftist and LGBT activist named Keaton Hill assaulted and threatened to kill @FJtheDeuce, a black conservati...

## Event detection methods and techniques

**Fig. 2:**
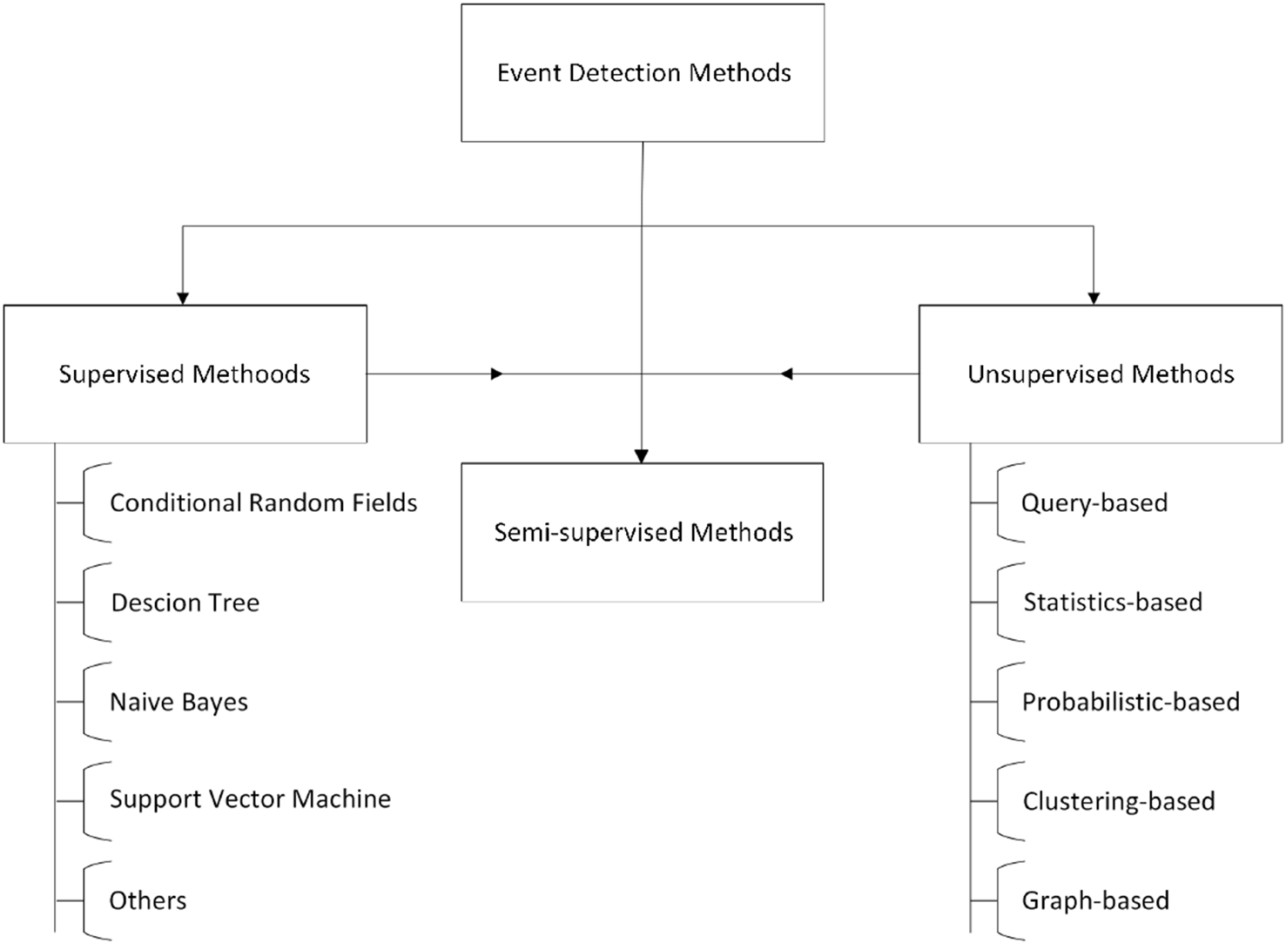
Classification of ED methods

Event Detection has been a popular topic in the research community. Several methods and techniques have been proposed to detect events depending on different requirements. These methods directly depend on the type of task and the data available. As such, they were detecting events from image data is undoubtedly different from text data. However, this work only refers to event detection techniques related to text data, particularly data obtained from social media platforms (Fig. 2).

Event detection methods and techniques revolve around a few basic approaches. Two approaches that are being used in event detection are document-pivot and feature-pivot. These approaches differ mainly in the clustering approach, the way documents are used to feature vectors, and the similarity metric used to identify if the two documents represent the same event. Another approach is the topic modeling approach, primarily based on probabilistic models.

**Fig. 3:**
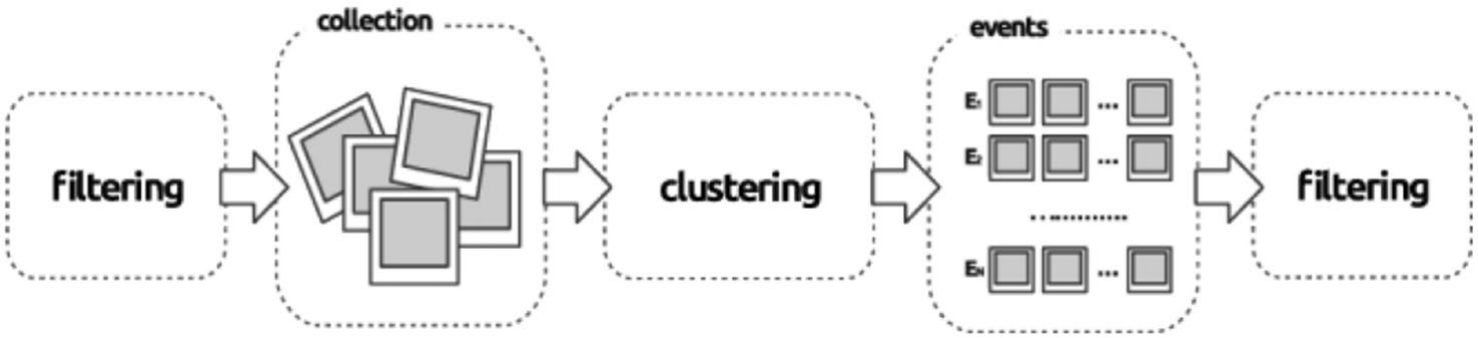
Event detection using document-pivot approach (Schinas et al. 2018)

It originates from the Topic Detection and Tracking task (TDT) field and can be seen as a clustering issue. *Document-pivot approach* detects events by clustering documents based on document similarity as given in Fig. 3. Documents are compared using cosine similarity with Tf-IDF (term frequency-inverse document frequency) representations, while a Locality Sensitive Hashing (LSH) (Datar et al. 2004) scheme is utilized to retrieve the best match rapidly.

**Fig. 4:**
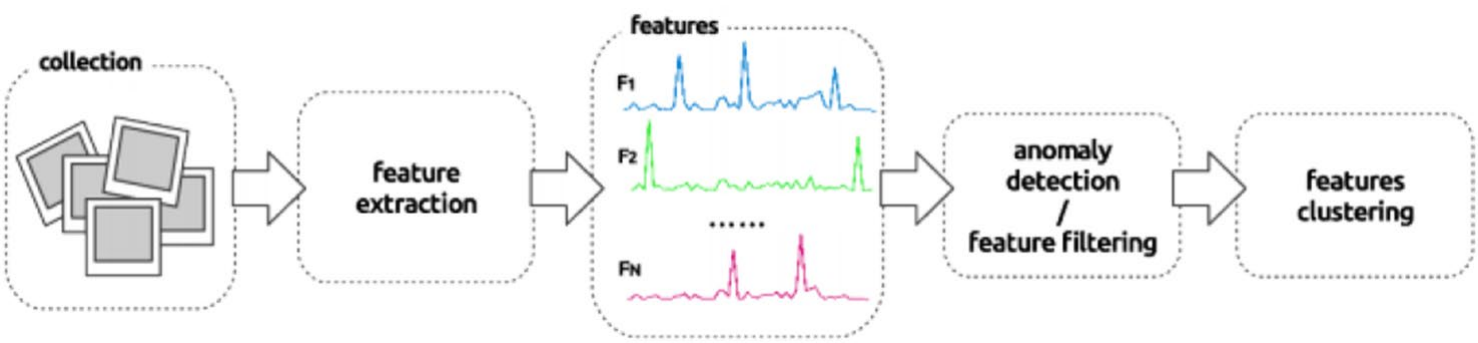
Event detection using feature-pivot approach (Schinas et al. 2018)

This technique was initially proposed for the analysis of timestamped document streams. The bursty activity is considered an event that makes some text features more prominent. The features can be keywords, entities, and phrases. *Feature-pivot Approach* clusters together with terms based on the pattern they occur as shown in Fig. 4. A study (Hossny et al. 2020) uses a Naive Bayes classifier to learn the selected features such as keywords to identify civil unrest and protests and accordingly predict the event days.

**Fig. 5:**
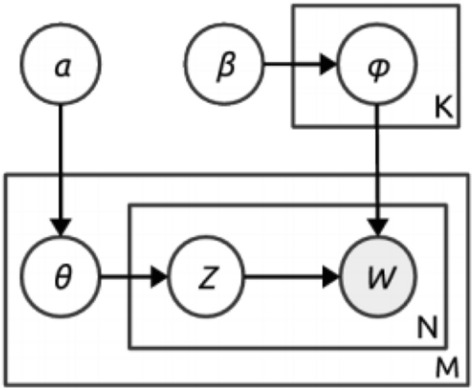
LDA—A common topic modeling technique (Schinas et al. 2018)

*Topic modeling approaches* are based on probabilistic models which detect events in social media documents in a similar way that topic models identify latent topics in text documents. In the beginning, topic models depended on word occurrence, where the text corpora were given as a mixture of words with latent model topics, and the set of identified topics was given as documents. Latent Dirichlet Allocation (LDA) (Jelodar et al. 2019) is the most known probabilistic topic modeling technique. It is a hierarchical Bayesian model where a topic distribution is supposed to have a sparse Dirichlet prior. The model is shown in Fig. 5, where $\alpha$ is the parameter of the Dirichlet before the per-document topic distribution $\vartheta$ and $\varphi$ is the word distribution for a topic. K represents the number of topics, M represents the document number, and N gives the number of words in a document. If the word W is the only observable variable, the learning of topics, word probabilities per topic, and the topic mixture of each document are tackled as a problem of Bayesian inference solved by Gibbs sampling.

Many methods are proposed for the detection of events. These event detections (ED) methods are mainly categorized under the category of supervised and unsupervised, as shown in Fig. 2. Supervised methods include support vector machine (SVM), Conditional random field (CRF), Decision tree (DT), Naive Bayes (NB), and others. At the same time, the unsupervised approaches include query-based, statistical-based, probabilistic-based, clustering-based, and graph-based.

### Supervised methods

Supervised methods are expensive and lengthy as they require labels and training, and this becomes difficult for more extensive data where the cost of training the model is higher and time-consuming. Some of the supervised methods for event detection are discussed below.

#### Support vector machines (SVM)

Support vector machines are based on the principle of minimizing structural risks (Vapnik 1995) of computer learning theory. Minimizing structural risks is to finding an assumption h for which we can guarantee the lowest true error. The real error in h is the probability that h will make an error in a sample test selected at random. An upper limit can be used to connect the true error of a hypothesis h with the error of h in the training set and the complexity of H (measured by VC-Dimension), the space of hypotheses which contains h (Vapnik 1995). The supporting vector machines find the hypothesis h, which (approximately) minimizes this limit on the true error by controlling effectively and efficiently the VC dimension of Joachims (1998).

It has been confirmed in many works that SVM is one of the most efficient algorithms for text classification. The accuracy of 87% was achieved to classify the traffic or nontraffic events on Twitter. It was able to identify valuable information regarding traffic events through Twitter (Salas et al. 2017). SVM combination with incremental clustering technique was applied to detect social and real-world events from photos posted on Flicker site (Sundaram and HaX 2012).

#### Conditional random fields (CRF)

The CRFs are an essential type of machine learning model developed based on the Maximum Entropy Markov Model (MEMM). It was first proposed by Lafferty et al. (2001) as a probabilistic model to segment and label sequence data, inherit the advantages of the previous models, increase their efficiency, overcome their defects, and solve more practical problems. A conditional Random Field (CRF) classifier was learned to extract the artist name and location of music events from a corpus of tweets (Benson et al. 2011).

#### Decision tree (DT)

Decision tree learning is a supervised machine learning technique for producing a decision tree from training data. A decision tree is also referred to as a classification tree or a reduction tree, and it is a predictive model which draws from observations about an item to conclusions about its target value. In the tree structure, leaves represent classifications (also referred to as labels), non-leaf nodes are features, and branches represent conjunctions of features that lead to the classification (Tan 2015). A decision tree classifier called gradient boosted was used to anticipate whether the tweets consist of an event concerning the target entity or not.

#### Naïve Bayes (NB)

Naïve Bayes is a simple learning algorithm that uses the Bayes rule and a strong assumption that the attributes are conditionally independent if the class is given. Although this independence assumption is often violated in practice, naïve Bayes often provides competitive accuracy. Its computational efficiency and many other distinctive features result in naïve Bayes being extensively applied in practice.

Naïve Bayes gives a procedure for using the information in sample data to determine the posterior probability P(y|x) of each class y, given an object x. Once we have such estimates, they can be used for classification or other decision support applications (Webb and Sammut 2010).

### Unsupervised methods

The unsupervised method does not usually require training or target labels. However, they can depend on specific rules based on the model and requirements. The unsupervised methods being used for event detection are discussed below. Scientists develop many unsupervised methods and are grouped into different categories described in the following subsections.

#### Query based methods

Query-based methods are based on queries and simple rules to identify planned rules from multiple websites, e.g., YouTube, Flicker, and Twitter. An event’s temporal and spatial information was extracted to inquire about other social media websites to obtain relevant information (Becker et al. 2012). The query-based method requires predefined keywords if there are many keywords to avoid unimportant events.

#### Statistical based methods

Different researchers under this category introduced many methods. For example, the average frequency of unigrams was calculated to find the significant unigrams (keywords) and combine those unigrams to illustrate the trending events. [29] The attempt was made to detect the hot events by identifying burst features (i.e., unigram) during different time windows. Each unigram bursty feature signal was then converted into a frequency domain. They were using Discrete Fourier Transformation (DFT). However, DFT could not detect the period when there is a burst which is very important in ED process (Subasic and Berendt 2011).

#### Wavelet transformation(WT)

Another technique called Wavelet Transformation (WT) was introduced to assign signals to each unigram feature. WT technique differs from DFT in terms of isolating time and frequency and provides better results (Wens and Sung Lee 2011). A new framework was proposed that integrated different unsupervised techniques. For example, LDA, NER, bipartite graph clustering algorithm based on relation and centrality scores to discover hidden events and extract their essential information such as time, location, and people that have been involved (Vavliakis et al. 2013).

#### Named entity relation(NER)

Named Entity Relation (NER) identifies increasing weights for the proper noun features. A proposed technique applied tweet segmentation to get the sentences containing more phrasing words instead of unigrams. Later, they computed the TFIDF of these sentences and user frequency and increased weights for the proper noun features identified by Named Entity Relation (NER). Li et al. (2012) first applied tweets and classified them using K-Nearest Neighbor (KNN) to identify the events from tweets published by Singapore users.

Weiler et al. (2014) used shifts of terms computed by Inverse Document Frequency (IDF) over a simple sliding window model to detect events and trace their evolution. Petrovic et al. (2010) modified and used Locality Sensitive Hashing (LSH) to perform First Story Detection (FSD) task on Twitter.

#### Probabilistic based methods

**Fig. 6:**
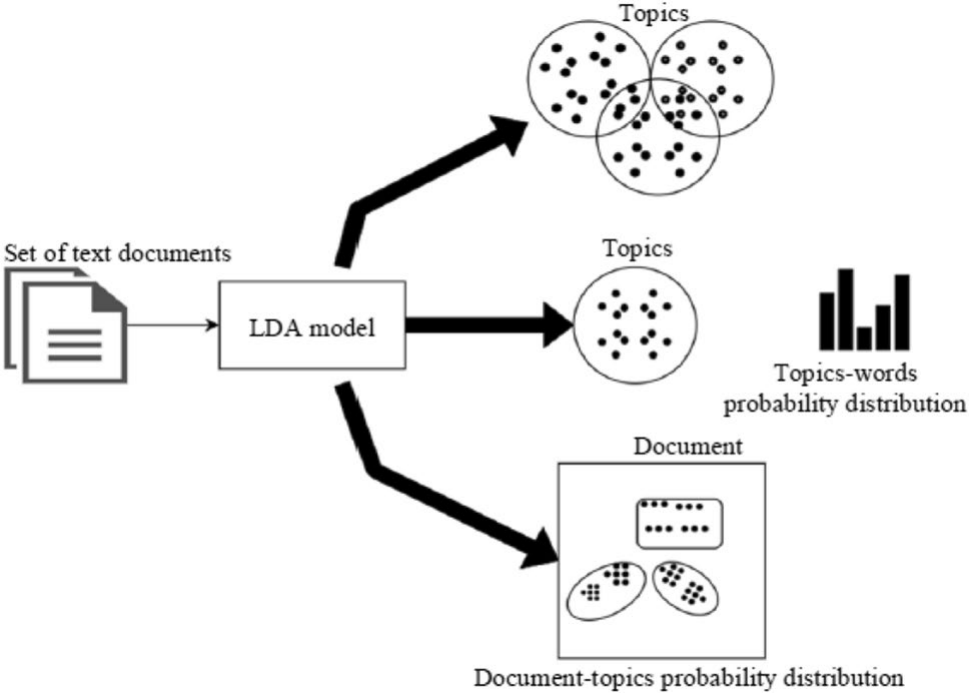
Topic modeling in LDA (AL-Dyani et al. 2020)

Latent Dirichlet Allocation (LDA) and Probabilistic Latent Semantic Indexing (PLSI) is topic modeling methods used for event detection. In LDA, each document has many topics, and each document should have a group of topics. The model is shown in Fig. 6.

LDA worked well with news articles and academic abstracts but fell short for small texts. However, the LDA model has been improved by adding tweet pooling schemes and automatic labeling. Pooling schemes include basic scheme, author scheme, burst term scheme, temporal scheme, and hashtag scheme tweets published under the same hashtag. The experiment results proved that the hashtag scheme produced the best cluster results (Mehrotra et al. 2013). However, LDA defines the number of topics and terms per topic in advance, inefficiently implementing it over social media.

#### Clustering-based method

**Fig. 7:**
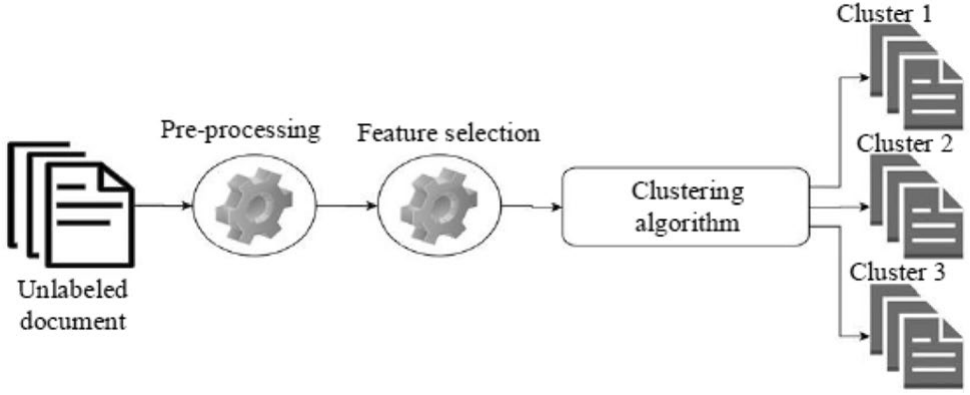
Clustering-based method (AL-Dyani et al. 2020)

Clustering-based methods mainly rely on selecting the most informative features, which contribute to event detection, unlike supervised methods, which need labeled data for prediction. It contributes to detecting events more accurately (Fig. 7).

Many clustering-based methods exist for text data, and K-means is a famous clustering algorithm. A novel dual-level clustering was proposed to detect events based on news representation with time2vec (Yu and Wu 2018). Clustering-based methods have been employed in various ways and other techniques such as NER, TFIDF, and others in different tasks, but the ideal clustering technique is still yet to come.

#### Graph-based methods

**Fig. 8:**
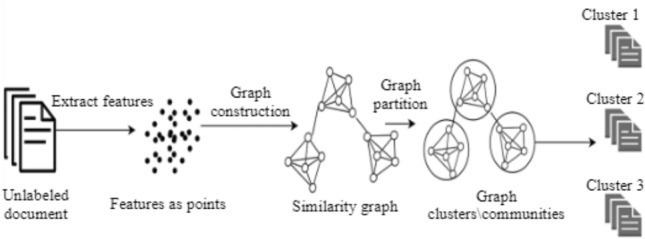
Graph-based clustering method (AL-Dyani et al. 2020)

Graph-based methods consist of nodes/vertices representing entities and edges representing the relationship between the nodes. Valuable information can be extracted from these graphs by grouping a set of nodes based on the set of edges. Each generated group is called a cluster/graph structure, a community, cluster, or module. The links between different nodes are called intra-edges. Meanwhile, links that connect different communities are called inter-edges (Fig. 8).

### Semi-supervised methods

Semi-supervised learning combines both supervised and unsupervised learning methods. Typically, a small number of labeled and largely unlabeled data is used for training purposes. Sometimes they are also referred to as the hybrid method. If there is a vast number of unlabeled data combined with insufficient labeled data, it can affect the classification accuracy. It is also referred to as imbalanced training data.

Similarly, the classification can become inefficient and accurate if there is no labeled data for a particular class. Semi-supervised methods include self-training, generative models, and graph-based methods. A semi-supervised algorithm based on tolerance roughest and ensemble learning is recommended for such kinds of problems (Shi et al. 2011). The missing class is extracted by approximation from the dataset and used as the labeled sample. The ensemble classifier iteratively builds the margin between positive and negative classes to estimate negative data further since negative data is mixed with positive data. Therefore, classification is done without training samples by applying a hybrid approach, and it saves the cost of getting labeled data manually, especially for larger datasets.

### Transformer-based pre-trained models

In 2017, Google Research published an article titled “Attention is all you need” that introduced the network architecture called transformers (Vaswani et al. 2017). The transformers solely rely on attention mechanisms to draw global dependencies between input and output while eschewing recurrence and convolutions models. Recent works establish that the transformer-based pre-trained models (PTMs) can achieve state-of-the-art performance in almost every NLP task (Qiu et al. 2020). The advancement of these models started with Generative Pre-trained Transformer (GPT) (Radford et al. 2018) and Bidirectional Encoder Representations from Transformers (BERT) (Devlin et al. 2018). These models are built on top of transformers architecture, self-supervised learning, and transfer learning. Generally, these models fall under semi-supervised learning for natural language. Some of the commonly used transformer models are briefly discussed below.

*BERT* is developed to pre-train deep bidirectional representations from the unlabeled text by jointly conditioning the left and right contexts. Its pre-trained model acts as the mind, which can then master and regulate the growingly large resources of discoverable content and queries and can be fine-tuned to the user’s specifications. This process is called transfer learning. The pre-trained BERT model can be fine-tuned with a single additional output layer to build state-of-the-art models for various NLP problems. BERT is pre-trained on an extensive corpus of unlabeled text, including Wikipedia (2500 million words) and books. As the model is trained on a large text corpus, the model begins to gain an in-depth and intimate conception of how the language works. BERT takes an input of a sequence of up to 512 tokens and outputs the sequence representation. The sequence has one or two segments, where the first token of the sequence is always [CLS] and contains the specific classification embedding, and another special token [SEP] is used to divide the segments. BERT arranges the final hidden state h of the first token [CLS] for text classification tasks to render the complete sequence. A softmax classifier is added to the top of the BERT model to get the predicted probabilities from the trained model. The data set must be vectorized to feed it to the classifier since it is originally in text format. BERT learns contextual embedding rather than context-free, such as in the case of Word2Vec. Although different models are available for text vectorization, BERT performs tokenization using the WordPiece method (Wu et al. 2016). BERT trains both Masked language modeling (MLM) and NSP objectives simultaneously. MLM is a self-supervised pretraining task extensively used in natural language processing for learning text representations. MLM trains a model to predict a random sample of input tokens that have been replaced by a [MASK] placeholder in a multi-class setting over the entire vocabulary (Yamaguchi et al. 2021). A recently published study shows how BERT can efficiently classify extreme negative sentiments in the context of extremism (Jamil et al. 2022). CrisisBERT particularly deals with the important task of crisis detection under the classification tasks of crisis detection, and crisis recognition (Liu et al. 2021). Different variations of BERT are proposed for specific tasks such as SpanBERT, DistilBERT, and RoBERTa (Joshi et al. 2019; Sanh et al. 2019; Liu et al. 2019).

*RoBERTa* stands for Robustly optimized BERT approach (Liu et al. 2019) which Facebook introduces. It is a retraining of BERT with improved training methodology, relatively more data, and computing power. The implementation of RoBERTa is the same as the Bert model with a small embedding tweak and a setup for Roberta pre-trained models. It has the same architecture as BERT but uses a byte-level pair encoding (BPE) tokenizer similar to GPT-2 and uses a different pretraining scheme. In particular, RoBERTa is trained with dynamic masking, FULL-SENTENCES without NSP loss, large mini-batches, and a larger byte-level BPE. To refine the training process, RoBERTa takes out the Next Sentence Prediction (NSP) task from BERT’s pre-training and introduces dynamic masking so that the masked token changes during the training epochs. The experiment also showed that the larger batch training sizes were also found to be more useful in the training procedure. Importantly, in addition to BERT training 16GB of Books Corpus and English Wikipedia data, RoBERTa uses 160 GB of text for pre-training. The additional data includes the CommonCrawl News dataset (63 million articles, 76 GB), Web text corpus (38 GB), and Stories from Common Crawl (31 GB). This, combined with a massive 1024 V100 Tesla GPU’s running for a day, resulting in pre-training of RoBERTa. Consequently, RoBERTa outperforms both BERT and XLNet (Yang et al. 2019) on GLUE benchmark results. RoBERTa has been proven effective on a variety of tasks such as detection of mental illnesses (Murarka et al. 2020), offensive language detection (Tanase et al. 2020), protest event detection (Re et al. 2021) etc.

*XLNet* (Yang et al. 2019) model is an extension of the Transformer-XL model (Dai et al. 2019). It is pre-trained using an autoregressive method like OpenGPT (Radford et al. 2018) and bi-directional context modeling of BERT by maximizing the anticipated likelihood over all permutations of the input sequence factorization order. OpenGPT Transformer learns using left-to-right text representation for natural language generation, while BERT uses a bidirectional transformer for natural language understanding. XLNet is a generalized autoregressive (AR) language modeling method that uses a permutation language modeling objective to combine the advantages of AR and autoencoding (AE) methods. The XLNet neural architecture is built to work effortlessly and harmoniously with the AR objective, including integrating Transformer-XL and carefully designing the two-stream attention mechanism. BERT is an Autoencoding (AE) based model, while XLNet is an Auto-Regressive (AR) that uses permutation language modeling. The permutation operation during pre-training allows the context to include to kens from both left and right, making it a generalized order-aware autoregressive language model. The proposed XLNet architecture is pre-trained using nearly ten times more data than the original BERT. It is also trained with a batch size eight times larger for half as many optimization steps, thus making it four times more sequences in pretraining than BERT. XLNet achieves substantial improvement over previous pretraining objectives on various tasks. It is claimed that the XLnet outperforms BERT on 20 tasks, often by a large margin. The findings of a study show that XlNet achieves slightly better results for potentially harmful and protective suicide-related content on Twitter (Metzler et al. 2022). Another study employed BERT, RoBERTa, XLNet, and seven other transformer-based models to find the victims of disasters on Twitter for the purpose of rescue operations (Zhou et al. 2022).

## Discussion

This section discusses different works related to event detection categorized under the types proposed earlier in this work. The types of events are scenario-based, sentiment-based, and action-based dangerous events. Each work is described in this section and its event type and technique. Furthermore, this section also discusses the research related to event prediction. Table 1 illustrates different type of events detection from social media.

**Table 1:** Dangerous Events categorized under relevant types

	Event type	Technique	References	Dataset	Years
Scenario-based					
	Natural disasters	SVM/SGD	Nourbakhsh et al. (2017)	Twitter	2017
	Earthquake	Classification(SVM)	Sakaki et al. (2010)	Twitter	2010
	Crisis	CrisisBERT	Liu et al. (2021)	Twitter (C6,C36)	2021
	Earthquake &Hurricane	Unsupervised	Arachie et al. (2019)	Twitter	2019
	Fire and Haze disaster	Classification (hotspots)	Kibanov et al. (2017)	NASA &Twitter	2017
	Emergency	Text-CNN, Linear SVC & Clustering	Huang et al. (2021)	Weibo	2021
	.	.	.	.	.
Sentiment-based					
	Extreme sentiments	Unsupervised learning	Pais et al. (2020)	misc.	2020
	COVID-19 sentiments	word2vec	Abdukhamidov et al. (2021)	Twitter & Instagram	2021
	Hate speech & offensive Language	BERT	Plaza-del-Arco et al. (2021)	HASOC(Twitter)	2021
	Far-right extremism	Classification	Kong et al. (2021)	Facebook, Twitter &Youtube	2021
	Political polarization	Clustering	Demszky et al. (2019)	Twitter	2019
	.	.	.	.	.
Action-based					
	Cyber attack	Unsupervised	Khandpur et al. (2017)	Twitter	2017
	Coordinated campaigns	Unsupervised	Pacheco et al. (2020)	Misc	2021
	Riots	Clustering	Ng et al. (2021)	Parler	2021
	Drugs Trafficking	SpanBERT	Zhu and Bhat (2021)	Text Corpus(subreddit)	2021
	Human Trafficking	Classification (NSI)	Yang et al. (2018)	Wiebo	2018
	.	.	.	.	.

### Detection of different/dangerous events on social media

Event detection has been long addressed in the Topic Detection and Tracking (TDT) in academia (Allan et al. 1998). It mainly focuses on finding and following events in a stream of broadcast news stories shared by social media posts. Event Detection (ED) is further divided into two categories depending on the type of its task; New Event Detection (NED) and Retrospective Event Detection (RED) (Li et al. 2005). NED focuses on detecting a newly occurred event from online text streams, while RED aims to discover strange events from offline historical data. Often event detection is associated with identifying the first story on topics of interest through constant monitoring of social media and news streams. Other related fields of research are associated with event detection, such as event tracking, event summarization, and event prediction. Event tracking is related to the development of some events over time. Event summarization outlines an event from the given data, while the event forecasts the next event within a current event sequence. These topics are part of the Topic Detection and Tracking (TDT) field.

Nourbakhsh et al. (2017) address natural and artificial disasters on social media. They identified events from local news sources that may become global breaking news within 24 h. They used Reuters News Tracer, a real-time news detection and verification engine. It uses a fixed sphere decoding (FSD) algorithm to detect breaking stories in real-time from Twitter. Each event is shown as a cluster of tweets engaging with that story. By considering different data features, they applied an SGD and SVM classifier that detects breaking disasters from postings of local authorities and local news outlets.

Sakaki et al. (2010) leverage Twitter for detecting earthquake occurrence promptly. They propose a method to scrutinize the real-time interaction of earthquake events and detect a target event similarly. Semantic analyses were deployed on tweets to classify them into positive and negative classes. The target for classification is two keywords; earthquake or shaking, which are also addressed as query words. Total of 597 positive samples of tweets that report earthquake occurrence are used as training data. They also implemented filtering methods to identify the location and an application called the earthquake reporting system in Japan.

Liu et al. (2021) aim for crisis events. They propose a state-of-the-art attention-based deep neural network model called CrisisBERT to embed and classify crisis events. It consists of two phases which are crisis detection and crisis recognition. In addition, another model for embedding tweets is also introduced. The experiments are conducted on C6 and C36 datasets. According to the authors, these models surpass state-of-the-art performance for detection and recognition problems by up to 8.2% and 25.0%, respectively.

Arachie et al. (2019) proposed an unsupervised approach for detecting sub-events in major natural disasters. Firstly, noun-verb pairs and phrases are extracted from tweets as an important sub-event prospect. In the next stage, the semantic embedding of extracted noun-verb pairs and phrases is calculated and then ranked against a crisis-specific ontology called management of Crisis (MOAC). After filtering these obtained candidate sub-events, clusters are formed, and top-ranked clusters describe the highly important sub-events. The experiments are conducted on Hurricane Harvey and the 2015 Nepal Earthquake datasets. According to the authors, the approach outperforms the current state-of-the-art sub-event identification from social media data.

Forests fire have become a global phenomenon due to rising droughts and increasing temperatures, often attributed to global warming and climate change. The work (Kibanov et al. 2017) tests the usefulness of social media to support disaster management. However, the primary data for dealing with such incidents come from NASA satellite imagery. The authors use GPS-stamped tweets posted in 2014 from Sumatra Island, Indonesia, which experiences many haze events. As confirmed by analyzing the dataset, Twitter has proven to be a valuable resource during such events. Furthermore, the authors also announced the development of a tool for disaster management.

Huang et al. (2021) focus on emergency events. They consider the various type of events under the term “emergency events”. It includes infectious diseases, explosions, typhoons, hurricanes, earthquakes, floods], tsunamis, wildfires, and nuclear disasters. To respond in time, the model must automatically identify the attribute information 3W (What, When, and Where) of emergency events. Their proposed solution contains three phases, the classification phase, the extraction phase, and the clustering phase, and it is based on the Similarity-Based Emergency Event Detection (SBEED) framework. The experiment is done using the Weibo dataset. Different classification models such as KNN, Decision Trees, Naïve Bayes, Linear SVC (RBF), and Text-CNN are used in the classification phase. Secondly, time and location are extracted from the classification obtained. Lastly, an unsupervised dynamical text clustering algorithm is deployed to cluster events depending on the text-similarity of type, time, and location information. The authors claim superiority of the proposed framework having good performance and high timeliness that can be described what emergency, and when and where it happened.

Pais et al. present an unsupervised approach to detecting extreme sentiments on social networks. Online wings of radical groups use social media to study human sentiments engaging with uncensored content to recruit them. They use people who show sympathy for their cause to further promote their radical and extreme ideology. The authors developed a prototype system composed of two components, i.e., Extreme Sentiment Generator (ESG) and Extreme Sentiment Classifier (ESC). ESG is a statistical method used to generate a standard lexical resource called ExtremesentiLex, containing only extreme positive and negative terms. This lexicon is then embedded in ESC and tested on five different datasets. ESC finds posts with extremely negative and positive sentiments in these datasets. The result verifies that the posts previously classified as negatives or positives are, in fact, extremely negatives or positives in most cases.

The COVID-19 pandemic has forced people to change their lifestyles, and Lockdown further pushed people to use social media to express their opinions and feelings. It provides a good source for studying users’ topics, emotions, and attitudes discussed during the pandemic. The authors of work (Abdukhamidov et al. 2021) collected two massive COVID-19 datasets from Twitter and Instagram. They explore data with different aspects, including sentiment analysis, topic detection, emotions, and geo-temporal. Topic modeling on these datasets with distinct sentiment types (negative, neutral, positive) shows spikes in specific periods. Sentiment analysis detects spikes in specific periods and identifies what topics led to those spikes attributed to economy, politics, health, society, and tourism. Results showed that COVID-19 affected significant countries and experienced a shift in public opinion. Much of their attention was on China. This study can be very beneficial to read people’s behavior in the aftermath; Chinese people living in those countries also faced discrimination and even violence because of the COVID-19 linked with China.

Plaza-del-Arco et al. (2021) investigate the link between hate speech and offensive language(HOF) with relevant concepts. Hate speech targets a person or group with a negative opinion, and it is related to sentiment analysis and emotion analysis as it causes anger and fear inside the person experiencing it. The approach consists of three phases and is based on multi-task learning (MTL). The setup is based on BERT, a transformer-based encoder pre-trained on a large English corpus. Four sequence classification heads are added to the encoder, and the model is fine-tuned for multi-class classification tasks. The sentiment classification task categorizes tweets into positive and negative categories, while emotion classification classifies tweets into different emotion categories (anger, disgust, fear, joy, sadness, surprise, enthusiasm, fun, hate, neutral, love, boredom, relief, none). The offense target is categorized as an individual, group, and unmentioned to others. Final classification detects HOF and classifies tweets into HOF and non-HOF.

Kong et al. (2021) explore a method that explains how extreme views creep into online posts. Qualitative analysis is applied to make ontology using Wikibase. It proceeded from the vocabulary of annotations such as the opinions expressed in topics and labeled data collected from three online social networking platforms (Facebook, Twitter, and Youtube). In the next stage, a dataset was created using keyword search. The labeled dataset is then expanded using a looped machine learning algorithm. Two detailed case studies are outlined with observations of problematic online speech from the Australian far-right Facebook group. Using our quantitative approach, we analyzed how problematic opinions emerge. The approach exhibits how problematic opinions appear over time and how they coincide.

Demszky et al. (2019) highlight four linguistic dimensions of political polarization in social media: topic choice, framing, and affect an apparent force. These features are quantified with existing lexical methods. The clustering of tweet embeddings is proposed to identify important topics for analysis in such events. The method is deployed on 4.4 M tweets related to 21 mass shootings. Evidence proves the discussions on these events are highly polarized politically, driven by the framing of biased differences rather than topic choice. The measures in this study provide connecting evidence that creates a big picture of the complex ideological division penetrating public life. The method also surpasses LDA-based approaches for creating common topics.

While most typical use of social media is focused on disease outbreaks, protests, and elections, Khandpur et al. (2017) explored social media to uncover ongoing cyber-attacks. The unsupervised approach detects cyber-attacks such as breaches of private data, distributed denial of service (DDOS) attacks, and hijacking accounts while using only a limited set of event trigger as a fixed input.

Coordinated campaigns aim to manipulate and influence users on social media platforms. Pacheco et al. (2020) work aim to unravel such campaigns using an unsupervised approach. The method builds a coordination network that relies on random behavioral traces between accounts. A total of five case studies are presented in the research, including U.S. elections, Hong Kong protests, the Syrian civil war, and cryptocurrency manipulation. Networks of coordinated Twitter accounts are discovered in all these cases by inspecting their identities, images, hashtag similarities, retweets, or temporal patterns. The authors propose using the presented approach for uncovering various types of coordinated information warfare scenarios.

Coordinated campaigns can also influence people towards offline violence. Ng et al. (2021) investigate the case of capital riots. They introduce a general methodology to discover coordinated by analyzing messages of user parleys on Parler. The method creates a user-to-user coordination network graph prompted by a user-to-text graph and a similarity graph. The text-to-text graph is built on the textual similarity of posts shared on Parler. The study of three prominent user groups in the 6 January 2020 Capitol riots detected networks of coordinated user clusters that posted similar textual content supporting different disinformation narratives connected to the U.S. 2020 elections.

Zhu and Bhat (2021) studies the specific case of the use of euphemisms by fringe groups and organizations that is expression substituted for one considered to be too harsh. The work claims to address the issue of Euphemistic Phrase detection without human effort for the first time. Firstly the phrase mining is done on raw text corpus to extract standard phrases; then, word embedding similarity is implemented to select candidates of euphemistic phrases. In the final phases, those candidates are ranked using a masked language model called SpanBERT.

Yang et al. (2018) explore the use of Network Structure Information (NSI) for detecting human trafficking on social media. They present a novel mathematical optimization framework that combines the network structure into content modeling to tackle the issue. The experimental results are proven effective for detecting information related to human trafficking.

Transfer learning is beneficial for various NLP tasks. However, negative transfer learning restricts the performance where the model solving an earlier problem makes later problems harder to solve. A study (Minoofam et al. 2021) proposes a transductive learning algorithm based on cellular learning automata (CLA) to deal with the issue of negative transfer (NT). The proposed algorithm leads to higher accuracy and fewer NT results.

Emotional speech can reveal vital information about the actual state of a person. However, fuzzy behavior can be a big hurdle while defining a person’s emotional state. To effectively overcome this issue, a study (Savargiv and Bastanfard 2013) investigates major challenges of designing and creating an emotional speech corpus. Another study (Hajarian et al. 2017) introduces a novel concept of fuzzy like and its two types implicit and explicit fuzzy like. It studies human behavior and shows how the social media audience can be reached effectively. This can reveal people’s tendency towards certain groups and ideologies.

Authors present Table 2 to clarify the intent of this work by providing an example of the collected tweets and their presumed techniques. Based on the existing methods for event detection, it gives a clear objective for using these methods for detecting dangerous events.

**Table 2:** Presumed types of dangerous events for tweets

Tweets	Proposed dangerous event type
“RT @KaitMarieox: This deranged leftist and LGBT activist named Keaton Hill assaulted and threatened to kill @FJtheDeuce, a black conservati...”	Action-based dangerous event
“RT @Lrihendry: When Trump is elected in 2020, I’m outta here. It’s a hate-filled sewer. It is nearly impossible to watch the hateful at...”	Sentiment-based dangerous event
“Scientists predict a tsunami will hit Washington, DC on 1/18/2020 We Are Marching in DC... https://t.co/3af4ZhyV3J”	Scenario-based dangerous event

### Event prediction

Event prediction is a complex issue that revolves around many dimensions. Various events are challenging to predict before they become apparent. For example, it is impossible to predict in case of natural disasters, and they can only be detected after the occurrence. Some events can be predicted while they are still in the evolving phase. Authors of Nourbakhsh et al. (2017) identify events from local news sources before they may become breaking news globally. The use case of COVID-19 can be regarded as an example where it started locally and became a global issue later.

A dataset is obtained from a recent Kaggle competition to explore the usability of a method for predicting disaster in tweets. The work in Chanda (2021) tests the efficiency of BERT embedding, an advanced contextual embedding method that constructs different vectors for the same word in various contexts. The result shows that the deep learning model surpasses other typical existing machine learning methods for disaster prediction from tweets.

Zhou et al. (2021) proposed a novel framework called Social Media enhAnced pandemic suRveillance Technique (SMART) to predict COVID-19 confirmed cases and fatalities. The approach consists of two parts; where firstly, heterogeneous knowledge graphs are constructed based on the extracted events. Secondly, a module of time series prediction is constructed for short-and long-term forecasts of the confirmed cases and fatality rate at the state level in the United States and finally discovering risk factors for intervening COVID-19. The approach exhibits an improvement of 7.3% and 7.4% compared to other state-of-the-art methods.

Incel behavior can cause violence and other extreme events in some cases. Detecting incel can help us prepare for the possible worse scenarios. A comprehensive study (Hajarian et al. 2019) investigates the profile of people inclined towards incel and provides a dataset for incel detection. Similarly, people inclined toward the extreme right, radical and criminal agenda can also help us predict the events based on the detected information.

Most of the other existing research targets particular scenarios of event prediction with limited scope. Keeping in mind the complexity of this problem, we only present a few related works, and the generalization is obscure.

### Event detection datasets

Due to the growth of the internet and related technologies, research in event detection has experienced significant interest and effort. However, the benchmark datasets for event detection witnessed slow progress. This can be attributed to the complexity and costliness of annotating events that require human input. There are a handful number of datasets available that covers event detection. These datasets are mostly limited to the small size of data and very restricted types of events. They address specific domains based on certain features. This also raises issues using a data-hungry deep learning model and typically requires balanced data for each class. Some of these datasets are briefed in the following paragraphs. Table 3 compares the discussed datasets and knowledge bases.

MAVEN (Wang et al. 2020) which stands for MAssive eVENt detection dataset, offers a general domain event detection dataset manually annotated by humans. It uses English Wikipedia and FrameNet (Baker et al., 1998) documents for building the dataset. It contains 111,611 various events and 118,732 events mentioned. The authors claim this to be the largest available human-annotated event detection dataset. There are 164 different events, representing a much wider range of public domain events. The event types are grouped under five top-level types: action, change, scenario, sentiment, and possession.

EventWiki (Ge et al. 2018) is a knowledge base of events, which consists of 21,275 events containing 95 types of significant events collected from Wikipedia. EventWiki gives four kinds of information: event type, event info-box, event summary, and full-text description. Authors claim to be the first knowledge base of significant events, whereas most knowledge bases focus on static entities such as people, locations, and organizations.

The EventKG (Abdollahi et al. 2020) is a multilingual1 resource incorporating event-centric information extracted from several large-scale knowledge graphs such as Wikidata, DBpedia, and YAGO, as well as less structured sources such as the Wikipedia Current Events Portal and Wikipedia event lists in 15 languages. It contains details of more than 1,200,000 events in nine languages. Supported languages include; English, French, German, Italian, Russian, Portuguese, Spanish, Dutch, Polish, Norwegian, Romanian, Croatian, Slovene, Bulgarian, and Danish.

EVIN (Kuzey et al. 2014) which stands for EVents In News, describes a method to extract events from a news corpus and organize them in relevant classes. It contains 453 classes of event types and 24,348 events extracted from f 300,000 heterogeneous news articles. The news articles used in this work are from a highly diverse set of newspapers and other online news providers (e.g., http://aljazeera.net/, http://www.independent.co.uk, http://www.irishtimes.com, etc.). These news articles were crawled from the external links mentioned on Wikipedia pages while ignoring the content of Wikipedia pages to get the articles from the original website source.

**Table 3:** Comparison of related event detection datasets

Dataset	Events	Event types	Document source	Language	Years	References
MAVEN	111, 611	164	English Wikipedia & FrameNet	English	2020	Wang et al. (2020)
EventWiki	21,275	94	English Wikipedia	English	2018	Ge et al. (2018)
EventKG	1,200,000	undefined	DBpedia & YAGO.	Multilingual(9)	2020	Abdollahi et al. (2020)
EVIN	24,348	453	news corpus	English	2014	Kuzey et al. (2014)

### Potential advantages and disadvantages

The concept of dangerous events is theoretical at this stage and a great outcome is expected. However, it can only be established after backing it with the results obtained after the experimentation. There are some potential advantages that can be considered at this stage are enlisted below:

- Creation of common base for all relates dangerous events.
- Discovering a general purpose method that can detect the majority of dangerous events.
- Construction of one of its kind comprehensive dangerous events dataset.
- Possible assistance in relating various dangerous events happening in real-time.
- Improving the ability to rank dangerous events according to the urgency of the immediate situation.

There are some foreseeable disadvantages associated with this approach that is enlisted below.

- Information suppression in case a larger number of dangerous events are detected.
- Generalization of different dangerous events that have distinct features.
- Encountering limitations of the model for detecting diverse kinds of events.
- Language constraint in case model is trained on specific language.

Various other advantages and disadvantages can be discussed better after learning the outcome of related experiments.

### Possible challenges

Many possible challenges can arise while detecting dangerous events. Some of the challenges are briefly discussed below.

#### Hybrid events

Hybrid events can happen both on the ground and virtually. In detecting such events, it is challenging to establish whether the detected event is a virtual or a live event. It adds further complexity, mainly if the classification method is used where the event will be classified according to the trained data model.

#### Establishing links between different events

This is another front in dangerous events to create the link between different events. As often happens in real life, one event can turn into another. One such scenario could be a peaceful protest turning into violence. Therefore, there is a need to build a mechanism for establishing the link between different events and the evolution of events.

#### Ranking based on priority

In case various dangerous events are detected, there is a need to prioritize the events according to their severity. As such, some dangerous events might be causing direct physical harm and need immediate intervention, while some could be just online bullying. Therefore, ranking the events according to their gravity is extremely important.

#### Time dimension

Time can reveal important information regarding the evolution of events, and it is a significant factor in predicting events. Hence, it is essential to introduce a time dimension in the method that can relate the events detected to provide insightful information. For example, an event may have occurred in the past, happened in the present, or planned to occur in the future. Based on that, further steps would be taken accordingly as per the requirements of the situation. The events occurring on social media may directly impact the personal or social life of the man/woman. Past events can tell us people’s opinions and other factors; current events can be a great source of developing a story, while future events can help us prepare in advance. The study (Dwarakanath et al. 2021) reviews the existing research for the detection of disaster events and classifies them into three dimensions early warning and event detection, post-disaster, and damage assessment.

#### Events dataset

There are few event datasets available. Many of these events are topic specific, and no known dataset combines all the dangerous events. Therefore, the need to build a general dangerous events dataset is crucial. One solution is to build a dataset using manual annotation. Since it is a lengthy and time-consuming process, it is proposed to build the dataset by filtering/combing existing datasets that can fall under the definition of dangerous events.

## Conclusion

Different methods exist for detecting various specific events on social media. In work, we proposed a new term using the analogy of “Dangerous Events” to unite all these events. Dangerous events contain a broad meaning, and they can be categorized based on certain similarities that exist among them. Categorizing dangerous events into sub-categories can help specify the event and its features. The proposed sub-categories consist of scenario-based, sentiment-based, and action-based dangerous events. The usefulness of social media these days provides a significant advantage in detecting such events in the early stages. While in some cases, significant events, also referred to as hybrid events, originate from social media and manifest in real life, such as mass protests, communal violence, and radicalization. Extreme events include extreme positive or extreme negative. However, dangerous events only fulfill extreme negative cases where there is a common wish to evade possible dangers posed to the safety of a person, group, or society. Detecting such dangerous events can ensure public safety while providing a broader view of the events happening. Various events happening in the virtual or real sphere are probably interrelated, while some might give rise to other dangerous events. Approaching the situation in a unified manner can give the advantage of prioritizing and acting in anticipation. Furthermore, it can lead to developing and discovering the best method for all such events. A dataset of dangerous events is crucial for the experiments, yet no specific dangerous event dataset exists. We believe there is an excellent scope for related work in the future. As a proposal, we suggest building a dataset containing all types of dangerous events by unifying all the related events to avoid the lengthy manual annotation process. Secondly, different techniques can be applied to this dataset to deepen the usefulness further and evolve a technique that can be generalized for all kinds of such events. Considering the limitations of event detection and techniques covering only specific events, a joint base can help discover the universally applicable method.

## Data Availability

Not applicable.

## Footnotes

1 http://sncrawler.di.ubi.pt/.

## Code availability

In the final version will be available code.
